# Comprehensive bioinformatics analysis to identify a novel cuproptosis-related prognostic signature and its ceRNA regulatory axis and candidate traditional Chinese medicine active ingredients in lung adenocarcinoma

**DOI:** 10.3389/fphar.2022.971867

**Published:** 2022-08-30

**Authors:** Shaohui Wang, Nan Xing, Xianli Meng, Li Xiang, Yi Zhang

**Affiliations:** ^1^ State Key Laboratory of Southwestern Chinese Medicine Resources, School of Ethnic Medicine, Chengdu University of Traditional Chinese Medicine, Chengdu, China; ^2^ State Key Laboratory of Southwestern Chinese Medicine Resources, School of Pharmacy, Chengdu University of Traditional Chinese Medicine, Chengdu, China; ^3^ State Key Laboratory of Southwestern Chinese Medicine Resources, Innovative Institute of Chinese Medicine and Pharmacy, Chengdu University of Traditional Chinese Medicine, Chengdu, China

**Keywords:** lung adenocarcinoma, cuproptosis, prognostic signature, DLD, ceRNA, ingredients

## Abstract

Lung adenocarcinoma (LUAD) is the most ordinary histological subtype of lung cancer, and regulatory cell death is an attractive target for cancer therapy. Recent reports suggested that cuproptosis is a novel copper-dependent modulated form of cell death dependent on mitochondrial respiration. However, the role of cuproptosis-related genes (CRGs) in the LUAD process is unclear. In the current study, we found that DLD, LIAS, PDHB, DLAT and LIPA1 in 10 differentially expressed CRGs were central genes. GO and KEGG enrichment results showed that these 10 CRGs were mainly enriched in acetyl-CoA biosynthetic process, mitochondrial matrix, citrate cycle (TCA cycle) and pyruvate metabolism. Furthermore, we constructed a prognostic gene signature model based on the six prognostic CRGs, which demonstrated good predictive potential. Excitedly, we found that these six prognostic CRGs were significantly associated with most immune cell types, with DLD being the most significant (19 types). Significant correlations were noted between some prognostic CRGs and tumor mutation burden and microsatellite instability. Clinical correlation analysis showed that DLD was related to the pathological stage, T stage, and M stage of patients with LUAD. Lastly, we constructed the lncRNA UCA1/miR-1-3p/DLD axis that may play a key role in the progression of LUAD and screened nine active components of traditional Chinese medicine (TCM) that may regulate DLD. Further, *in vitro* cell experiments and molecular docking were used to verify this. In conclusion, we analyzed the potential value of CRGs in the progression of LUAD, constructed the potential regulatory axis of ceRNA, and obtained the targeted regulatory TCM active ingredients through comprehensive bioinformatics combined with experimental validation strategies. This work not only provides new insights into the treatment of LUAD but also includes a basis for the development of new immunotherapy drugs that target cuproptosis.

## Introduction

Lung cancer is a serious threat to human health all over the world (Wang et al., 2018). Lung adenocarcinoma (LUAD), as the most common histological subtype of lung cancer, has a significantly increasing incidence compared with squamous cell carcinoma, large cell carcinoma, and small cell carcinoma (Meza et al., 2015; Ruiz-Cordero and Devine, 2020). The pathogenesis of LUAD is still not completely clear, and it is mostly due to a combination of lifestyle, environment, genetics, and other factors (Akhtar and Bansal, 2017). Currently, the most common treatments for LUAD include surgery, radiotherapy, drug therapy, and chemotherapy (Wu et al., 2021). Although early CT screening enables early detection and treatment of some patients with LUAD, the effect and prognosis of conventional treatment are not satisfactory due to the special invasiveness and drug resistance of LUAD (Rosell et al., 2020; Toki et al., 2020). In addition, there is an urgent need to identify and screen new prognostic markers and targeted drugs for LUAD in the face of many poor outcomes in patients with LUAD.

Recently, a new cell death pathway termed cuproptosis was found in addition to conventional cell death, such as apoptosis, pyroptosis, and ferroptosis (Tsvetkov et al., 2022). As a common trace metal element, copper plays an important role in maintaining multiple physiological functions of the human body, such as electron transfer, mitochondrial function, and the activities of various enzymes (Vetchý, 2018). Recent studies have shown that excess copper directly binds to lipoacylated proteins by mediating the tricarboxylic acid cycle and targets its upregulated factors, namely, the gene FDX1 that encodes the enzyme that reduces Cu^2+^ to Cu^1+^; excess copper also promotes the abnormal oligomerization of lipoacylated proteins, reduces protein lipoacylation, and reduces the level of Fe-S cluster proteins, resulting in copper-dependent cell death (Tsvetkov et al., 2019; Tsvetkov et al., 2022). The presence of copper ion carriers and glutathione consumption can promote copper-mediated cell death, whereas the presence of copper chelates can alleviate death to some extent. Studies have shown that after pulse treatment with copper ion carrier elesclomol, metabolites related to the tricarboxylic acid (TCA) cycle, such as citrate, cis-monucinic acid, and guanosine diphosphate, show time-dependent maladjustment. Moreover, if SLC31A1 is overexpressed in ABC-1 cells, the sensitivity of cells to copper is enhanced, resulting in cell copper death (Tsvetkov et al., 2022). Therefore, we can explore new therapeutic strategies for LUAD from the mechanism of cuproptosis to overcome the defects of traditional therapy.

The rapid development of multi-omics technology, artificial intelligence, and big data provides a powerful means to explore the development of tumors and potential therapeutic markers (Chakraborty et al., 2018; Hristova and Chan, 2019). Traditional Chinese medicine (TCM) has played an important role in tumor prevention and treatment, and the search for potential anti-tumor active ingredients in TCM has attracted the attention of scholars (Wang et al., 2021). This study aimed to elucidate the expression and prognostic significance of CRGs (cuproptosis-related factors) in LUAD through a comprehensive bioinformatics strategy, identify the potential regulatory axis of ceRNA of CRGs, and screen their targeted regulatory TCM active ingredients. In conclusion, this work can provide a sufficient basis to determine the prognostic value of CRGs in LUAD and develop cuproptosis-targeting modulators for the prevention and treatment of LUAD.

## Materials and methods

### Data collection and pretreatment

TCGA LUAD and GTEx corresponding normal tissue data were obtained. RNAseq data in TCGA and GTEx TPM format were processed by the Toil processes (Vivian et al., 2017) at UCSC XENA. The data included TCGA paracancer samples (59 cases), TCGA tumor tissue (515 cases), and GTEx normal samples (288 cases). RNAseq data in level 3 HTSeq-FPKM format from the TCGA LUAD project were obtained. Before further analysis, we converted the RNAseq data in fragments per kilobase per million (FPKM) formats to transcripts per million reads (TPM) format and log2 conversion.

### Acquisition, differential expression, gene mutation, and correlation analyses of CRGs

Ten CRGs, namely, FDX1, LIAS, LIPT1, DLD, DLAT, PDHA1, PDHB, MTF1, GLS, and CDKN2A, were obtained through an original research paper published in *Science* (Tsvetkov et al., 2022). R software (version 3.6.3) and Mann-Whitney *U* test (Wilcoxon rank-sum test) were further used to identify the differential expression of CRGs in LUAD and normal lung tissues. The Gene Set Cancer Analysis (GSCA) database (http://bioinfo.life.hust.edu.cn/GSCA/#/) was used to analyze the gene mutation of the 10 CRGs. We then constructed a protein-protein interaction (PPI) network of the 10 CRGs using the STRING database (https://cn.string-db.org/) and Cytoscape software (version 3.7.1).

### Enrichment analysis of gene ontology and kyoto encyclopedia of genes and genomes pathways

Gene ontology (GO) included biological processes (BP), cell composition (CC), and molecular function (MF) categories. The Kyoto encyclopedia of genes and genomes (KEGG) pathways were generated from the org. hs.eg.db package (version 3.10.0, for ID conversion), clusterprofiler package (version 3.14.3, for enrichment analysis) and ggplot2 (version 3.3.3, for visualization) packages in R software. The species was set as *Homo sapiens*, and p. adjust<0.1 and qvalue<0.2 were selected as screening conditions to obtain the main enrichment functions and pathways.

### Construction of cuproptosis-related prognostic gene signature model

Survminer package [version 0.4.9] (for visualization) and survival package [version 3.2–10] (for statistical analysis of survival data) in R software (version 3.6.3) were used to determine the 10 CRGs in predicting overall survival (OS) in LUAD, and *p* < 0.05 was considered statistically significant. Then we selected the CRGs with significant prognostic value for subsequent prognostic model construction. And the model formula is: risk score = Gene 1 expression value × α 1 + Gene 2 expression value × α 2 +... + Gene n expression value × α n. Where α is the regression coefficient calculated by the LASSO Cox regression analysis. Finally, The CRG prognostic model was constructed by LASSO Cox regression analysis, and the OS time difference between the two subgroups (low-risk subgroup and high-risk subgroup) was compared by Kaplan-Meier survival analysis. The prediction accuracy and risk score of each cuproptosis-related prognostic gene were compared by time ROC analysis. All the above analyses were performed by R software, and *p* < 0.05 was considered to be statistically significant.

### Analysis of immune invasion, tumor mutation burden, microsatellite instability, and drug sensitivity

The ssGSEA (GSVA embedded algorithm) (Hänzelmann et al., 2013) was used as the immune infiltration algorithm to analyze the correlation between the expression level of cuproptosis-related prognostic genes and the immune infiltration degree of 24 immune cell types (Bindea et al., 2013) and the enrichment score. Spearman correlation was used to analyze the relationship between cuproptosis-related prognostic genes and tumor mutation burden (TMB) and microsatellite instability (MSI) scores, and *p* < 0.05 was considered to be statistically significant. In addition, the GSCA database (http://bioinfo.life.hust.edu.cn/GSCA/#/) was used to analyze the drug sensitivity of cuproptosis-related prognostic genes.

### DLD expression verification and construction of ceRNA regulatory network

The human protein map (HPA) database (https://www.proteinatlas.org/) was used to validate the protein expression levels of DLD in normal lung tissue and LUAD. The StarBase database was used to predict DLD-relevant miRNA targets. Then, Mann-Whitney *U* test and Kaplan-Meier analysis were used to evaluate the expression and prognostic value of DLD related miRNAs in LUAD, and *p* < 0.05 were considered to be statistically significant. Then, we selected the miRNAs with significant differences as the research objects. Furthermore, the LncBase database and StarBase database were used to predict the lncRNA targets associated with miRNA. Subsequently, we adopted the same methods (Mann-Whitney *U* test and Kaplan-Meier analysis) to analyze the expression and prognostic value of these lncRNAs in the TCGA LUAD data set, and *p* < 0.05 was considered to be statistically significant. Finally, we determined and constructed a DLD related ceRNA regulatory network.

### Screening of TCM candidate effective ingredients for targeting the regulation of DLD

The CTD database (http://ctdbase.org/) was used to screen potential TCM chemical constituents targeting DLD, and Chemdraw (version 20.0) was used to map their structures.

### *In vitro* cell experiments validation

Normal lung epithelial cells (BEAS-2B) (Shanghai Zhong Qiao Xin Zhou Biotechnology Co., Ltd., Shanghai, China) and LUAD cell lines (H1299 and A549) (Hunan Fenghui Biotechnology Co., Ltd.) were maintained with DMEM (Gibco, ThermoFisher Scientific, Waltham, United States) containing 10% FBS (Gibco, ThermoFisher Scientific, Waltham, United States) and antibiotics and RPMI-1640 (Gibco, ThermoFisher Scientific, Waltham, United States) containing 10% FBS and antibiotics in 5% CO_2_ at 37°C, respectively. When the cell density reached more than 80%, it was used for subsequent experimental detection. According to the previous description (Wang et al., 2018), RNA was extracted and separated and their expression levels were detected by using RT-qPCR assays. The GAPDH was used as an endogenous control of lncRNA UCA1 and DLD, the U6 was used as an endogenous control of miR-1-3p, and the primers of lncRNA UCA1, miR-1-3p, DLD were shown in Supplementary Table S1. The experiment was repeated three times, and the data were expressed as mean ± standard deviation (SD). GraphPad Prism software (version 9.0, CA, United States) was used for statistical analysis, and ANOVA was used for comparison between multiple groups. *p* < 0.05 was considered statistically significant.

### Molecular docking verification

The 3D structures of nine potential active ingredients (resveratrol, genistein, aristolochic acid I, cannabidiol, epigallocatechin gallate, fructose, phlorizin, quercetin, and triptonide) were downloaded from PubChem database (https://pubchem.ncbi.nlm.nih.gov/). The 3D structure of DLD was downloaded from the PDB protein database (http://www.rcsb.org/pdb/home/home. do). Further, the protein was dehydrated and ligand extracted with PyMOL software. Then, Autodock software was used to conduct molecular simulation docking for the nine potential active ingredients and DLD, and the binding strength of DLD and the nine active ingredients was evaluated according to the docking binding energy.

## Results

### Analysis of GRP expression, gene mutation, and PPI in LUAD

**FIGURE 1:**
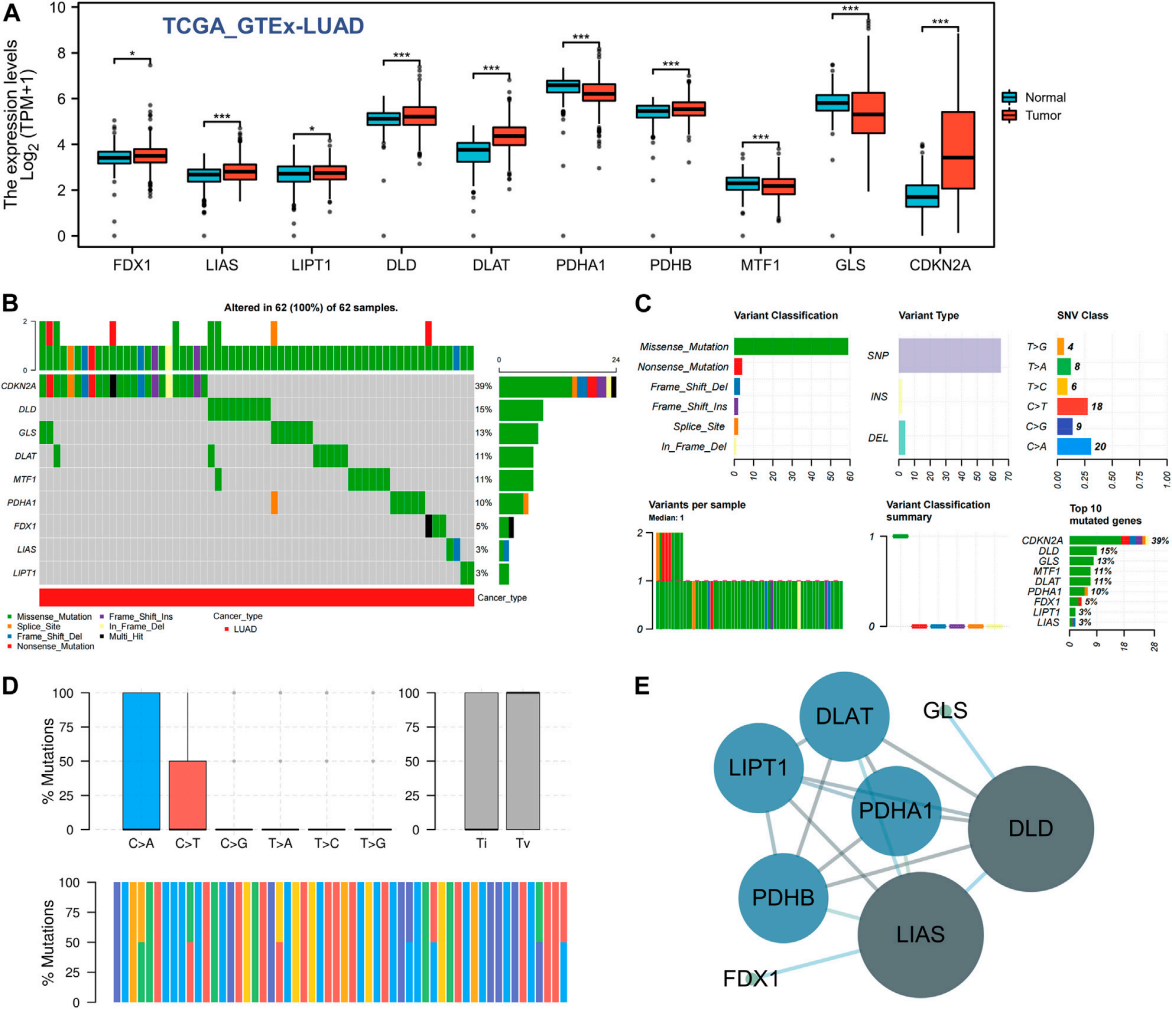
Expression difference and gene mutation of CRGs in LUAD. **(A)** Expression differences of 10 CRGs in LUAD and normal lung tissues. *, *p* < 0.05; ***, *p* < 0.001. **(B,C)** Gene mutation and classification of 10 CRGs in LUAD. **(D)** Transition (Ti) and transversion (Tv) classification of the SNVs of 10 CRGs in LUAD. **(E)** Interactive network of CRGs; the darker the color is, the larger the circle is, indicating that it belongs to the central target in the whole network.

We first used the TCGA GTEx-LUAD dataset to evaluate the expression of the 10 GRPs in LUAD and normal lung tissues, and the results showed that the mRNA levels of all 10 GRPs were changed in unpaired samples. Compared with the normal lung tissue, the expression levels of FDX1, LIAS, LIPT1, DLD, DLAT, PDHB, and CDKN2A in LUAD were upregulated, whereas the expression levels of PDHA1, MTF1, and GLS were downregulated (Figure 1A). We further analyzed the gene mutations of GRPs in LUAD, and the results revealed that all the 10 GRPs had gene mutations in LUAD samples, among which CDKN2A was the gene with the highest mutation rate, followed by DLD and GLS (Figure 1B). A missense mutation was the most common variation classification, SNP was the most common variant type, and C>A was listed as the top-class SNV (Figure 1C). Further classifying the mutations as transitions (Ti) and transversions (Tv), we found that Ti was generated at a higher frequency than Tv in the whole gene (Figure 1D). In addition, we constructed a PPI network of the 10 GRPs through the STRING database (Supplementary Figure S1) and found that DLD, LIAS, PDHB, DLAT, and LIPA1 were the central genes via Cytoscape (Figure 1E).

### GO and KEGG enrichment analyses of GRPs

**FIGURE 2:**
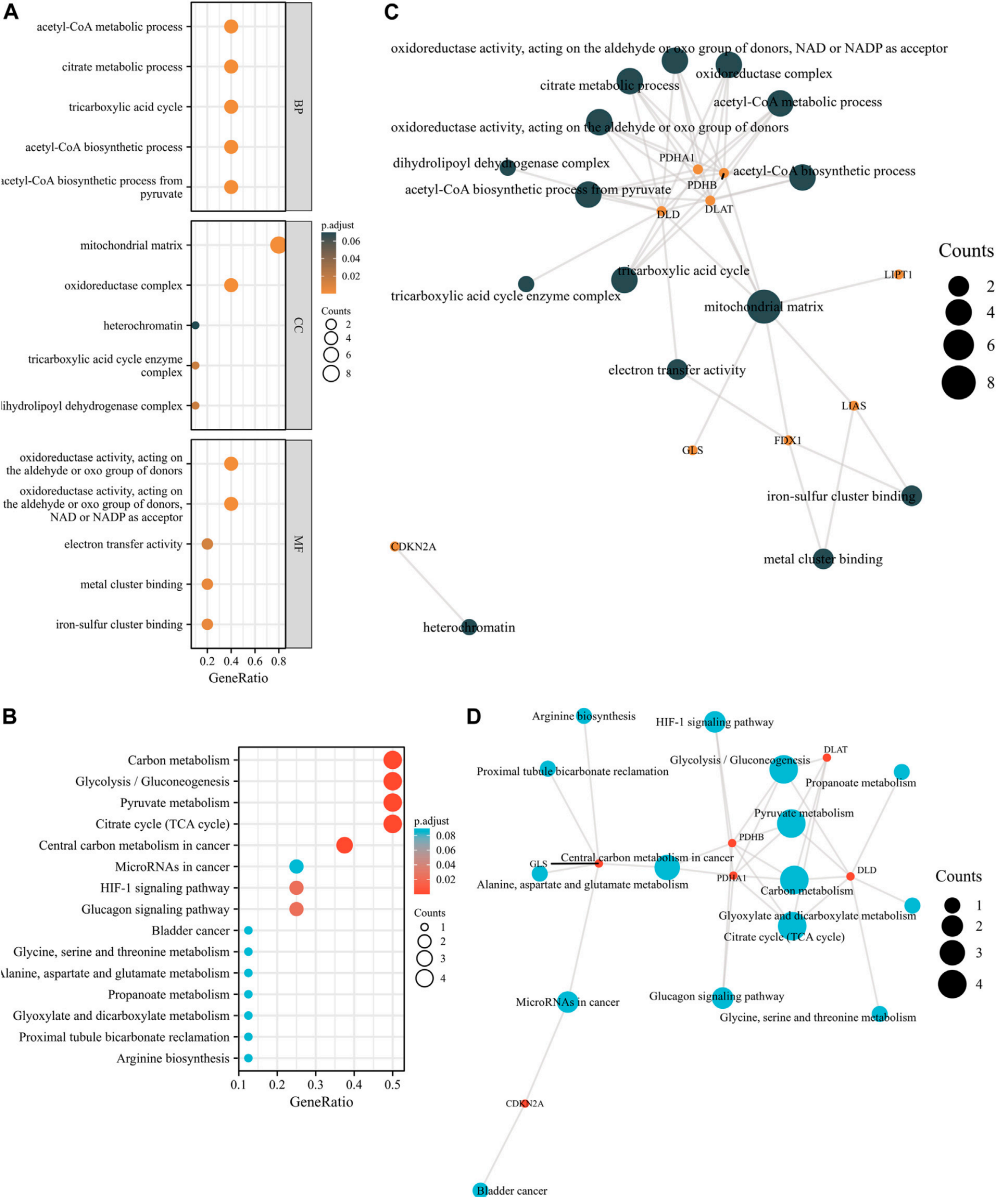
GO and KEGG enrichment analyses of 10 CRGs in LUAD. **(A)** Bubble plot of GO enrichment results of 10 CRGs in LUAD. **(B)** Bubble plot of KEGG enrichment results of 10 CRGs in LUAD. **(C)** Interactive network of GO entries and CRGs. **(D)** Interactive network of KEGG entries and CRGs.

To elucidate the potential function of these GRPs, we subsequently performed GO and KEGG pathway analyses of the 10 CRGs. The results showed that these 10 CRGs were mainly enriched in the acetyl-CoA biosynthetic process from pyruvate (GO:0006086), acetyl-CoA biosynthetic process (GO:0006085), mitochondrial matrix (GO:0005759), oxidoreductase complex (GO:1990204), oxidoreductase activity (GO:0016903), and metal cluster binding (GO:0051540) in GO functional analysis (Figure 2A). KEGG pathway analysis showed that the 10 CRGs participated in the citrate cycle (TCA cycle; hsa00020), pyruvate metabolism (hsa00620), glycolysis/gluconeogenesis (hsa00010), carbon metabolism (hsa01200), and central carbon metabolism in cancer (hsa05230) (Figure 2B). Furthermore, we found that four CRGs, namely, DLD, PDHA1, PDHB, and DLAT, had the highest degree values in the abovementioned enriched pathways and functions (Figures 2C,D).

### Construction of cuproptosis-related prognostic gene signature model

**FIGURE 3:**
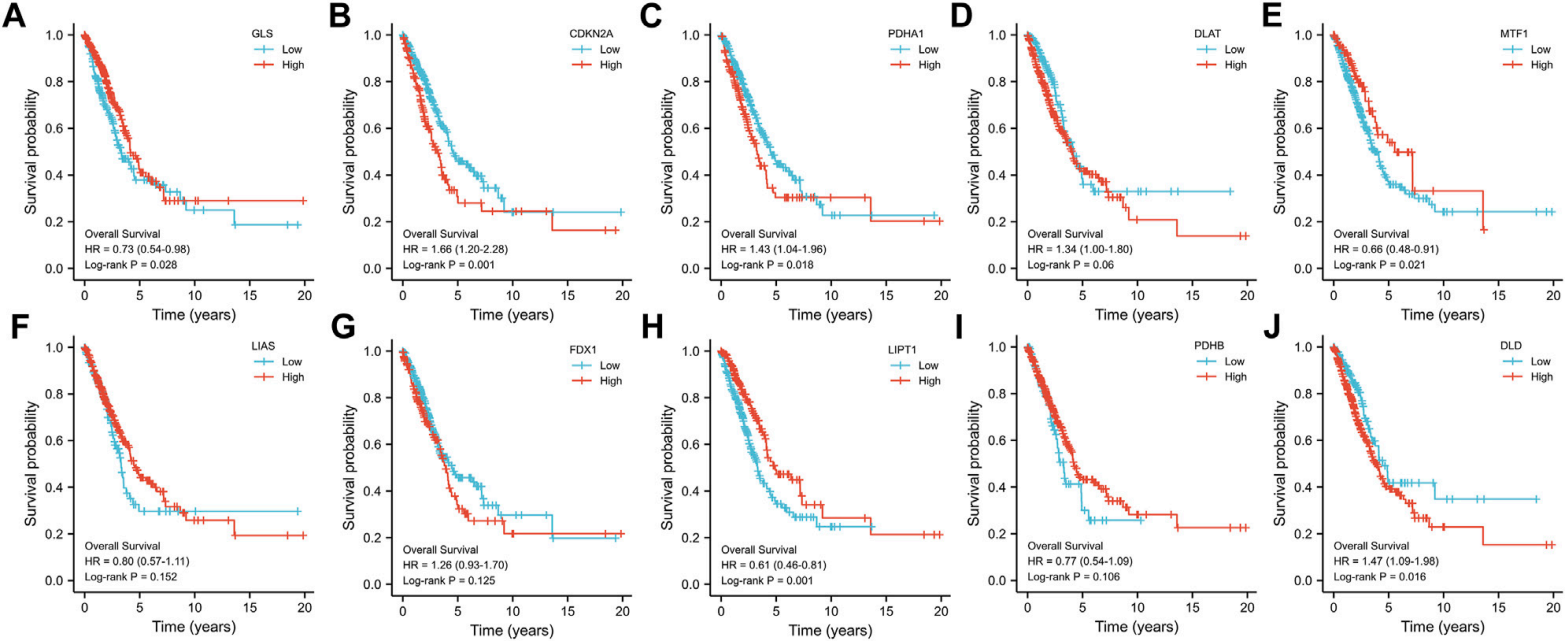
Prognostic value of 10 CRGs in LUAD. The OS curves of GLS **(A)**, CDKN2A **(B)**, PDHA1 **(C)**, DLAT **(D)**, MTF1 **(E)**, LIAS **(F)**, FDX1 **(G)**, LIPT1 **(H)**, PDHB **(I)**, and DLD **(J)** in patients with LUAD in the low and high expression groups.

**FIGURE 4:**
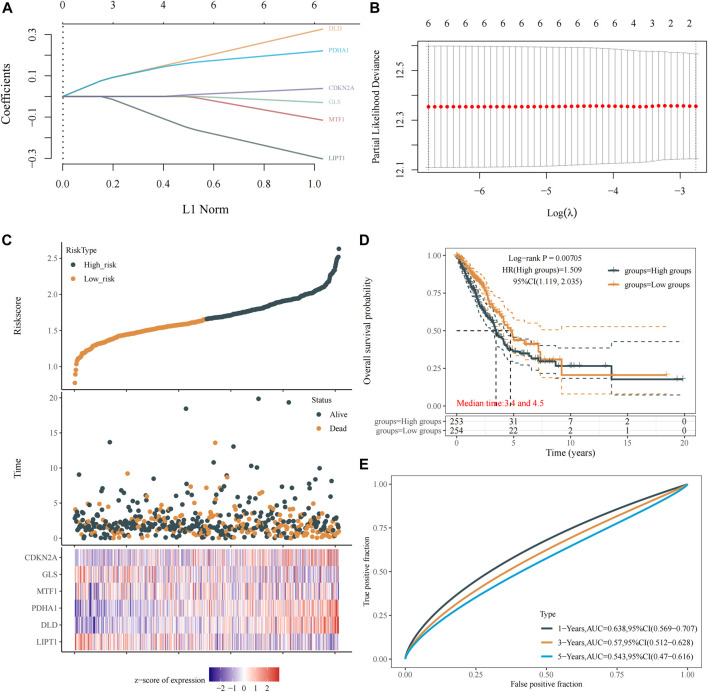
Construction of a prognostic signature model of CRGs in LUAD. **(A)** LASSO coefficients of the six prognostic CRGs. **(B)** PLD of the six prognostic CRGs. **(C)** Distribution of risk score, survival status, and expression of the six prognostic CRGs. **(D)** OS curve of patients with LUAD in the low and high expression groups. **(E)** 1-, 3-, and 5-year ROC prediction curves for patients with LUAD.

To construct the cuproptosis-related prognostic gene signature model, we conducted univariate Cox regression analysis to evaluate the prognostic value of these 10 differentially expressed CRGs. As shown in Figures 4A–J, we found that six CRGs were associated with the prognosis of patients, namely, GLS (Figure 3A), CDKN2A (Figure 3B), PDHA1 (Figure 3C), MTF1 (Figure 3E), LIPT1 (Figure 3H), and DLD (Figure 3J). On the basis of the six prognostic CRGs, we further constructed a cuproptosis-related prognostic gene signature model using LASSO Cox regression analysis. The risk score=(−0.3021)*LIPT1+(0.3259)*DLD+(0.2209)*PDHA1+(−0.1147)*MTF1+(−0.0294)*GLS+(0.0386)*CDKN2A. Figures 4A,B show the prognostic characteristic coefficients and partial likelihood bias of prognostic characteristics in patients with LUAD. Subsequently, we divided all patients with LUAD into low-risk and high-risk subgroups based on the risk score. Figure 4C shows the risk score, survival status, and expression of patients with LUAD. OS curve analysis showed that patients had a higher risk of death and a shorter survival time (*p* = 0.00705, median time = 3.4 vs. 4.5 years) with the increase in risk score (Figure 4D). ROC curves of risk models at different times were further analyzed, and the results showed that the areas under the ROC curve at 1, 3, and 5 years were 0.638, 0.57, and 0.543, respectively (Figure 4E).

### Correlation analysis of immune infiltration

**FIGURE 5:**
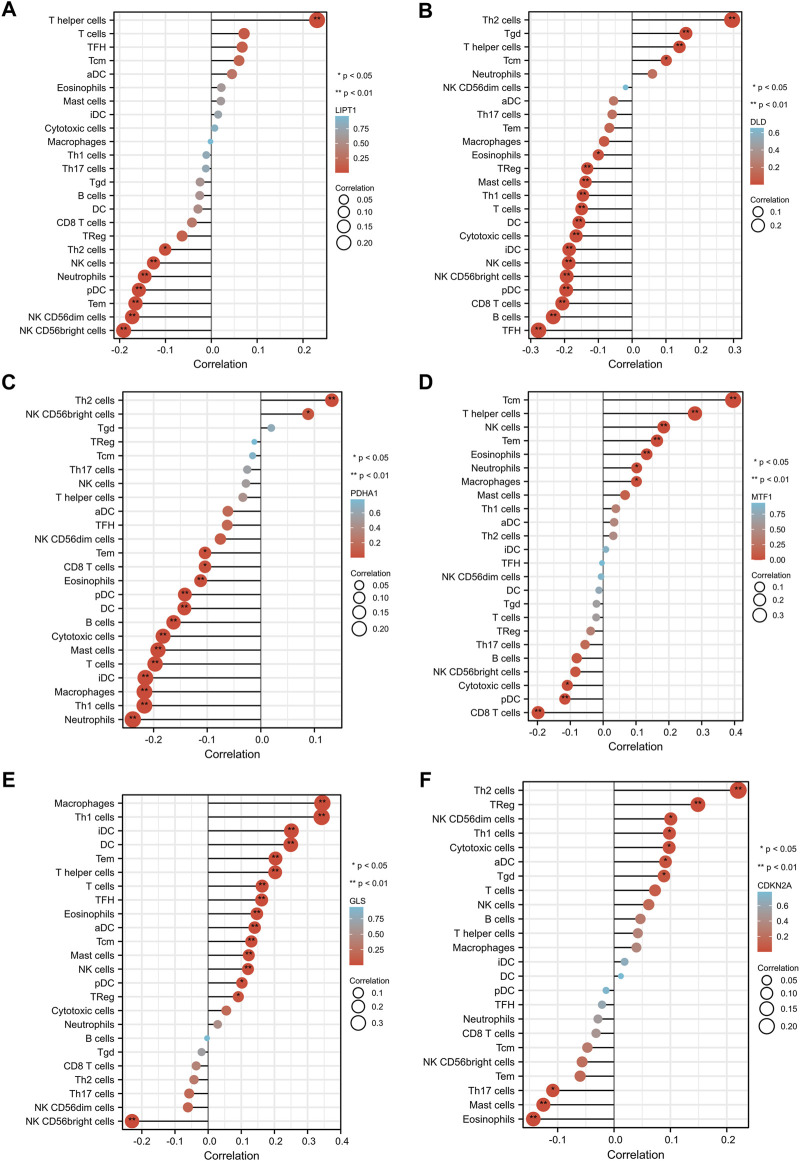
Correlation between the six prognostic CRGs and immune infiltration in LUAD. The correlation between LIPT1 **(A)**, DLD **(B)**, PDHA1 **(C)**, MTF1 **(D)**, GLS **(E)**, CDKN2A **(F)**, and the degree of immune infiltration of 24 immune cell types in patients with LUAD.

**FIGURE 6:**
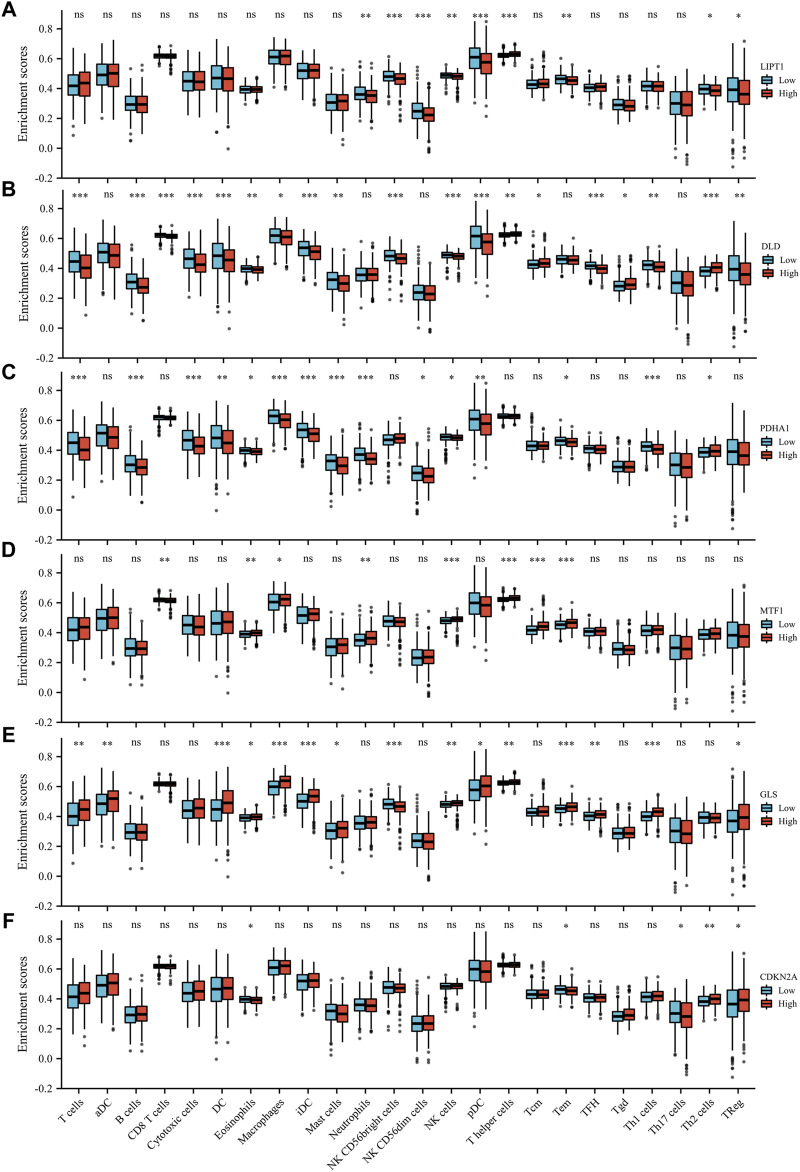
Enrichment scores of the six prognostic CRGs in 24 immune cell types in LUAD. The six prognostic CRGs were LIPT1 **(A)**, DLD **(B)**, PDHA1 **(C)**, MTF1 **(D)**, GLS **(E)**, and CDKN2A **(F)**.

We evaluated the correlation between the expression of prognostic CRGs (including LIPT1, DLD, PDHA1, MTF1, GLS, and CDKN2A) and the immune infiltration of 24 different immune cell types in LUAD. The results showed that LIPT1 expression was positively correlated with T helper cells. It was negatively correlated with NK CD56bright cells, NK CD56dim cells, Tem, pDC, neutrophils, NK cells, and Th2 cells (Figure 5A). DLD was positively correlated with Th2 cells, Tgd, T helper cells, and Tcm. By contrast, it was negatively correlated with TFH, B cells, CD8 T cells, pDC, NK CD56bright cells, NK cells, iDC, cytotoxic cells, DC, T cells, Th1 cells, mast cells, Treg, and eosinophils (Figure 5B). PDHA1 was positively correlated with Th2 cells and NK CD56bright cells, but it was negatively correlated with neutrophils, Th1 cells, macrophages, iDC, T cells, mast cells, cytotoxic cells, B cells, DC, pDC, eosinophils, CD8 T cells, and Tem (Figure 5C). MTF1 was positively correlated with Tcm, T helper cells, NK cells, Tem, eosinophils, neutrophils, and macrophages, but it was negatively correlated with CD8 T cells, pDC, and cytotoxic cells (Figure 5D). GLS was positively correlated with macrophages, Th1 cells, iDC, DC, Tem, T helper cells, T cells, TFH, eosinophils, aDC, Tcm, mast cells, NK cells, pDC, and Treg, but it was negatively correlated with NK CD56bright cells (Figure 5E). CDKN2A was positively correlated with Th2 cells, TReg, NK CD56dim cells, Th1 cells, cytotoxic cells, aDC, and Tgd, but it was negatively correlated with eosinophils, mast cells, and Th17 cells (Figure 5F). Further analysis showed that the enrichment types (*p* < 0.05) of LIPT1, DLD, PDHA1, MTF1, GLS, and CDKN2A in these 24 different immune cell types were 9 (Figure 6A), 19 (Figure 6B), 15 (Figure 6C), 8 (Figure 6D), 15 (Figure 6E), and 5 (Figure 6F), respectively. DLD was the most common, followed by PDHA1 and GLS. In conclusion, our results demonstrated a significant association between prognostic CRGs and lung tumor immune infiltration, and DLD was significantly enriched in most immune cell types.

### TMB, MSI, and drug sensitivity analyses

**FIGURE 7:**
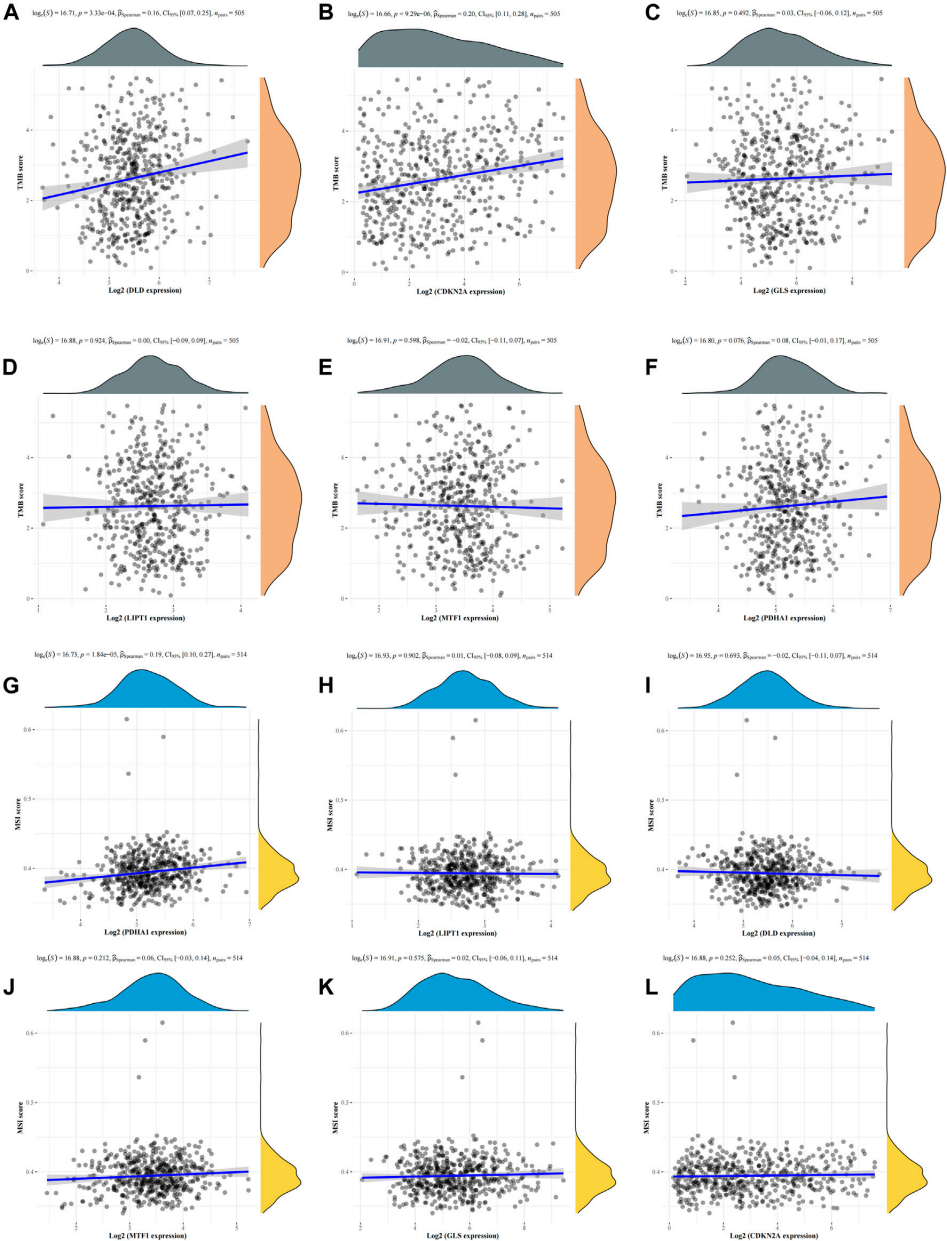
Correlation analysis of the six CRGs with TMB and MSI in LUAD. **(A–F)** Correlation between the six CRGs and TMB in LUAD. **(G–L)** Correlation between the six CRGs and MSI in LUAD.

To explore whether these six CRGs can also be used as biomarkers for drug screening, we subsequently analyzed the correlation between CRGs and TMB and MSI in LUAD. The results showed that DLD (Figure 7A) and CDKN2A (Figure 7B) were positively correlated with TMB, whereas GLS (Figure 7C), LIPT1 (Figure 7D), MTF1 (Figure 7E), and PDHA1 (Figure 7F) were not significantly correlated with TMB. In MSI analysis, only PDHA1 (Figure 7G) was found to be significantly positively correlated with MSI, whereas LIPT1 (Figure 7H), DLD (Figure 7I), MTF1 (Figure 7J), GLS (Figure 7K), and CDKN2A (Fig. 7L) were not significantly correlated with MSI. We further analyzed the relationship between the expression of the six CRGs and existing drugs. Drug sensitivity analysis showed that the expression of CDKN2A, DLD, LIPT1, and MTF1 was negatively correlated with most drugs in the GSCA database (Supplementary Figure S2).

### Clinical correlation analysis

**FIGURE 8:**
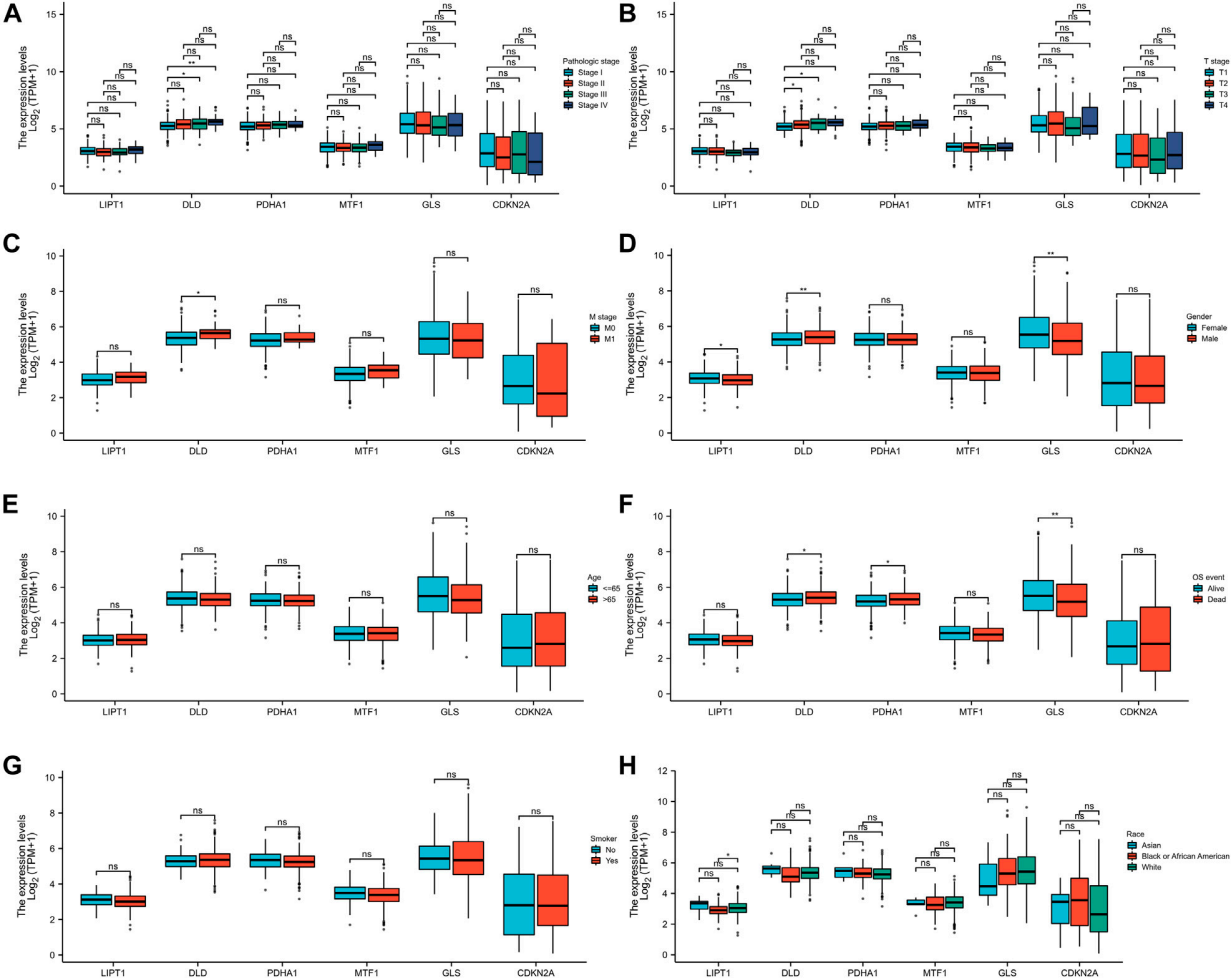
Association analysis of the six CRGs and different clinical parameters in patients with LUAD. The different clinical factors included pathologic stage **(A)**, T stage **(B)**, M stage **(C)**, gender **(D)**, age **(E)**, OS event **(F)**, smoker **(G)**, and race **(H)**.

We further evaluated the relationship between the expression levels of these six CRGs and different clinical parameters of patients with LUAD, and the results showed that DLD expression was related to the pathological stages (Stage Ⅰ, Stage Ⅲ, and Stage Ⅳ; Figure 8A), T stages (T1 and T3; Figure 8B), M stages (M0 and M1; Figure 8C), gender (Figure 8D), and OS (Figure 8F) of patients with LUAD, but it was not related to the changes in age (Figure 8E), smoke (Figure 8G), and race (Figure 8H). GLS expression was correlated with gender (Figure 8D) and OS (Figure 8F) but not with other factors. PDHA1 was only related to OS (Figure 8F). LIPT1 was only associated with race (Back or African American and White) (Figure 8H). MTF1 and CDKN2A were not clinically relevant (Figures 8A–H). These results fully proved that DLD may play an important role in the development of LUAD.

### Construction of the ceRNA regulatory network

**FIGURE 9:**
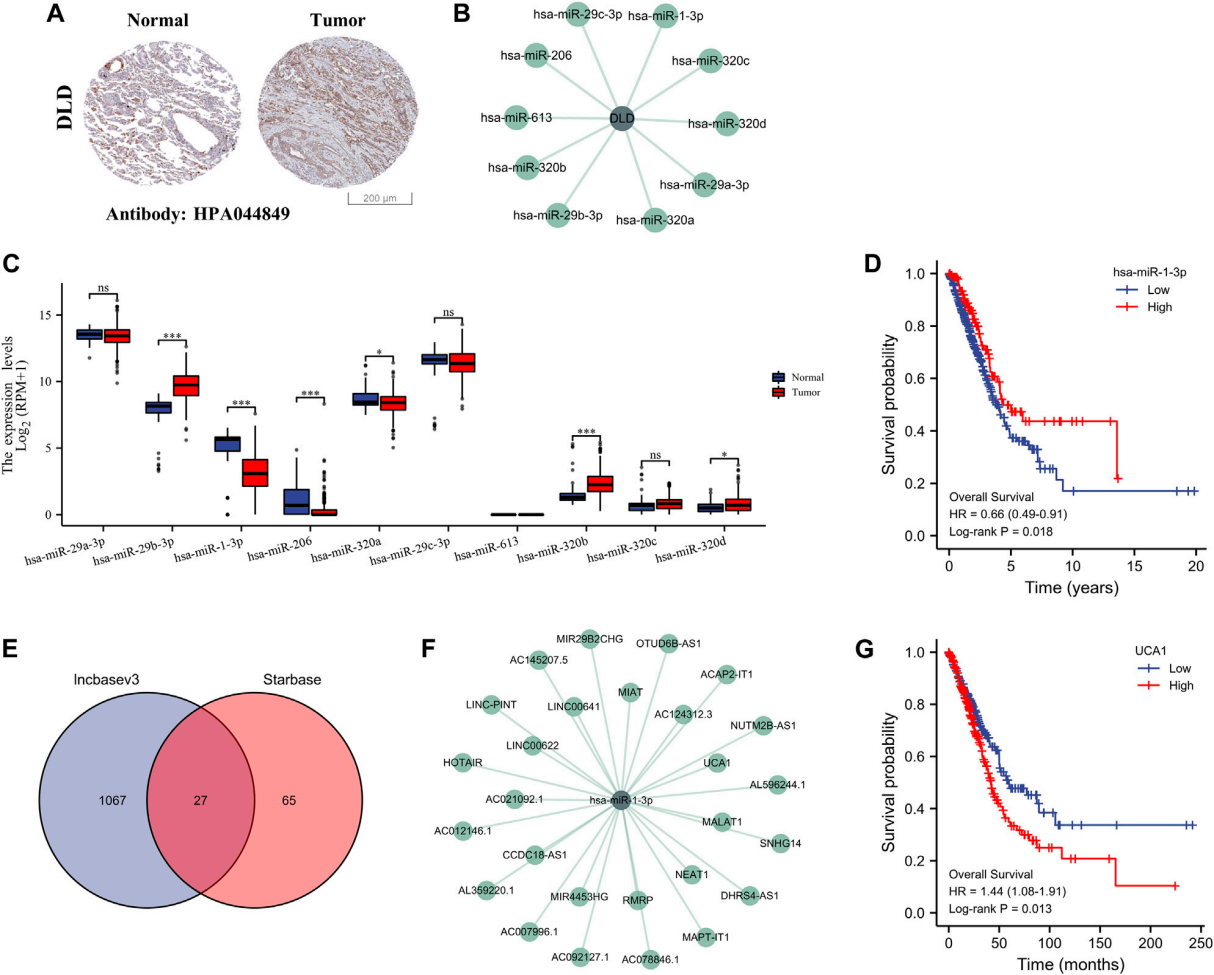
Construction of the ceRNA regulatory axis. **(A)** Differential expression of DLD protein in LUAD and normal lung tissues (HPA). **(B)** Ten miRNAs associated with DLD. **(C)** Differential expression of the 10 miRNAs in LUAD and normal lung tissues. ns, *p* ≥ 0.05; *, *p* < 0.05; ***, *p* < 0.001. **(D)** OS curves of DLD in patients with LUAD in the low and high expression groups. **(E)** Identification of 20 lncRNAs by the Starbase and lncbasev3 databases. **(F)** The 27 miRNAs associated with miR-1-3p. **(G)** OS curves of UCA1 in patients with LUAD in the low and high expression groups.

On the basis of the above screening results, we selected the most important pivotal gene DLD as the object of further study to fully explore its potential ceRNA network in the regulation of LUAD. We first verified the protein expression level of DLD using the HPA database, and the results showed that DLD was highly expressed in the tissues of patients with LUAD (Figure 9A). We predicted 10 potential miRNA targets of DLD using the StarBase database (Figure 9B) and evaluated the expression of these 10 miRNAs in LUAD samples. The results showed significant differences in the expression of six miRNAs in LUAD, namely, hsa-miR-29b-3p, hsa-miR-1-3p, hsa-miR-206, hsa-miR-320a, hsa-miR-320b, and hsa-miR-320d. Among them, hsa-miR-1-3p and hsa-miR-206 were significantly downregulated in LUAD (Figure 9C). Therefore, we further evaluated the prognostic value of these two miRNAs and found that only the expression of hsa-miR-1-3p was related to the prognosis of patients with LUAD, suggesting that patients with LUAD and high expression of miR-1-3p had a higher survival probability than their counterparts (Figure 9D). Therefore, miR-1-3p was considered the most promising miRNA target for DLD. In addition, 27 lncRNA targets related to miR-1-3p were predicted by the StarBase and LncBase databases (Figures 9E,F). Similarly, we detected the expression of these 27 lncRNAs in LUAD, and the results showed that 18 lncRNAs were expressed differently in LUAD. In particular, AC007996.1, AC021092.1, AC078846.1, CCDC18-AS1, HOTAIR, MIAT, MIR4453HG, and UCA1 were highly expressed in LUAD (Supplementary Figure S3). Further prognostic analysis showed that only UCA1 was significantly associated with the prognosis of patients with LUAD, and this result suggested that patients with LUAD and high UCA1 expression had a lower survival probability than their counterparts (Figure 9G, Supplementary Figure S4). Therefore, the lncRNA UCA1/miR-1-3p/DLD axis might play a key role in the progression of LUAD.

### Screening of TCM active ingredients targeting DLD

**FIGURE 10:**
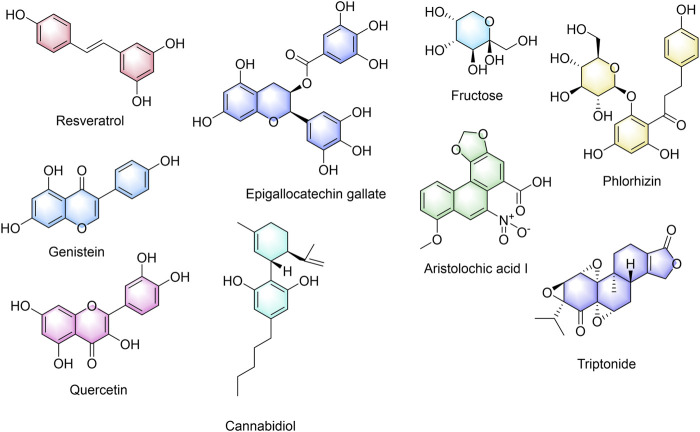
Active ingredients of traditional Chinese medicine with a potential effect on DLD.

To search for the potential TCM active components acting on cuproptosis prognosis-related gene DLD, we obtained nine chemical components of TCM that may be related to cuproptosis prognosis-related gene DLD by screening the CTD database. Figure 10 shows the nine active ingredients of TCM, namely, resveratrol, genistein, aristolochic acid I, cannabidiol, epigallocatechin gallate, fructose, phlorizin, quercetin, and triptonide.

### Experimental verification

**FIGURE 11:**
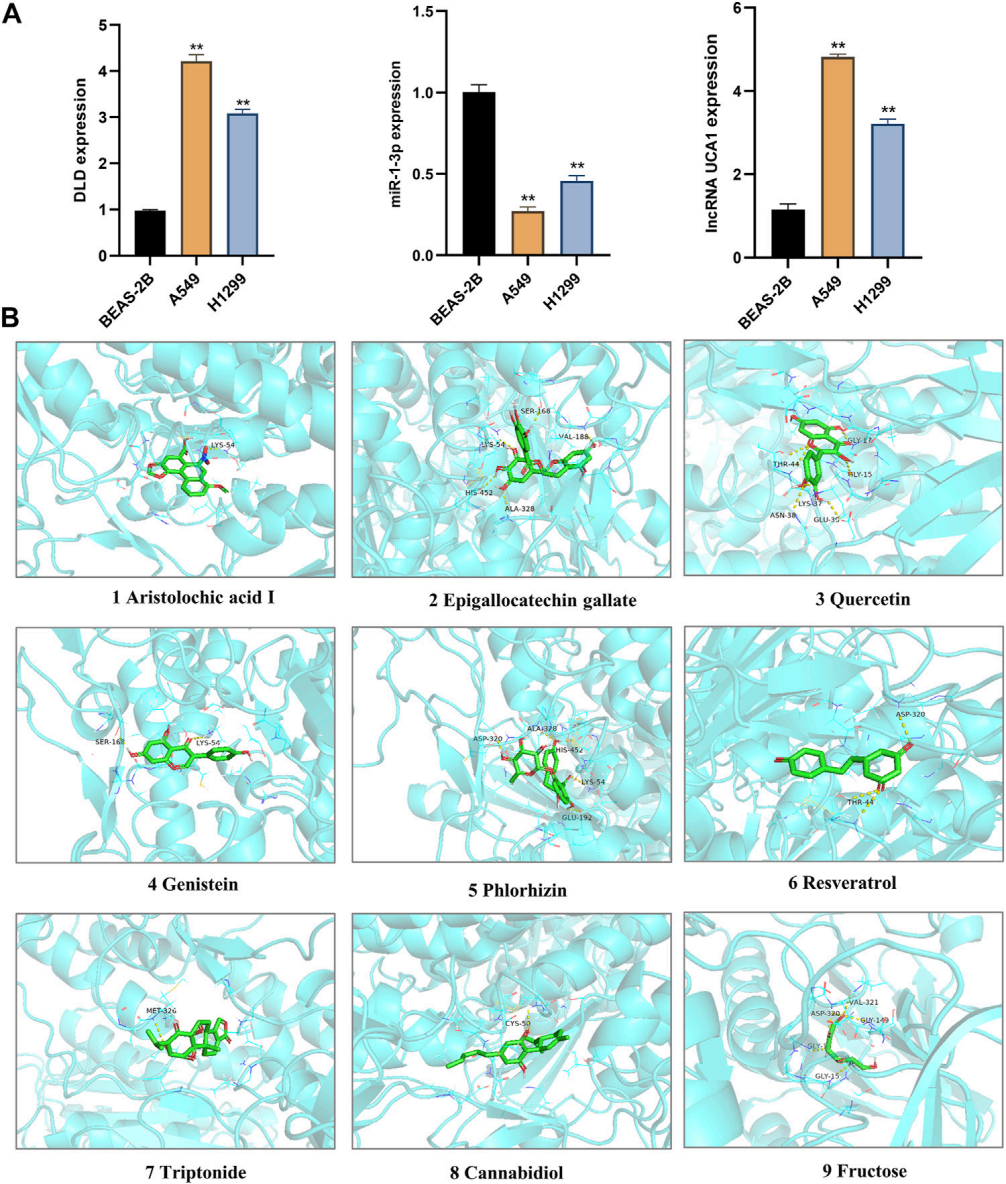
Validation of *In vitro* cell experiment and molecular docking. **(A)** The differential expression of lncRNA UCA1, miR-1-3p and DLD in normal lung epithelial cells (BEAS-2B) and different LUAD cell lines (A549 and H1299) were detected by *in vitro* cell experiments. **, *p* < 0.01. **(B)** The interaction between nine TCM active components and DLD protein was simulated by molecular docking.

To confirm the roles of lncRNA UCA1, miR-1-3p and DLD in LUAD, we further verified their differential expressions in normal lung epithelial cells (BEAS-2B) and different lung adenocarcinoma cell lines (A549 and H1299) by *in vitro* cell experiments. And the results showed that lncRNA UCA1 and DLD were significantly overexpressed in A549 and H1299 cell lines, and underexpressed in BEAS-2B cell lines, while miR-1-3p showed an opposite trend (Figure 11A). The above *in vitro* cell validation results were consistent with our bioinformatics prediction results. Meanwhile, we also verified the binding energy of these nine potential TCM chemical components to DLD by molecular docking technology, and the results were shown in Table 1. It is generally believed that the lower the binding energy of the ligand to the receptor, the greater the possibility of interaction between the ligand and the receptor. Our results showed that the binding energy of the nine predicted TCM active ingredients and DLD were all less than -5 kcal/mol, which fully proved the potential interaction between them. The molecular docking modes were shown in Figure 11B.

**TABLE 1:** The binding energy of nine TCM active components to DLD by molecular docking.

No.	Protein	PDB ID	Compound name	Binding energy (kcal/mol)
1	DLD	6I4R	Aristolochic acid I	−9.8
2			Epigallocatechin gallate	−9.7
3			Quercetin	−9.4
4			Genistein	−9.3
5			Phlorhizin	−9.2
6			Resveratrol	−8.7
7			Triptonide	−8.4
8			Cannabidiol	−8.2
9			Fructose	−5.6

## Discussion

We first clarified the expression of 10 GRPs in LUAD, and the results showed that the mRNA levels of these 10 GRPs were significantly different. Among them, seven GRPs, namely, FDX1, LIAS, LIPT1, DLD, DLAT, PDHB, and CDKN2A, were upregulated in LUAD. Three GRPs, namely, PDHA1, MTF1, and GLS, were downregulated in LUAD. Cancer is a collection of diseases characterized by abnormal and uncontrolled cell growth caused by genetic mutations. These mutations are called “drivers” after they drive tumorigenesis, and their form of mutation affects the homeostasis of a range of cell key functions (Martínez-Jiménez et al., 2020). Therefore, we conducted mutation analysis, and the results showed that CDKN2A was the gene with the highest mutation rate, followed by DLD and GLS. PPI networks are composed of proteins interacting with one another to participate in biological signal transmission, gene expression regulation, energy and substance metabolism, cell cycle regulation, and other life processes (Athanasios et al., 2017). By studying the interaction network between proteins, we can discover the core regulatory genes. Therefore, we analyzed the PPI network relationship among these 10 CRGs and found that DLD, LIAS, PDHB, DLAT, and LIPA1 were the core genes.

Through functional enrichment analysis, we found that these 10 CRGs were mainly involved in the citrate cycle (TCA cycle), pyruvate metabolism, glycolysis/gluconeogenesis, carbon metabolism, and other pathways. Some studies found that human non-small-cell lung cancers (NSCLCs) oxidize glucose in the TCA cycle (Faubert et al., 2017). Breast cancer cells rely on nutritional pyruvate to drive collagen-based remodeling of the extracellular matrix in lung metastases. Inhibition of pyruvate metabolism impairs collagen hydroxylation, thereby impairing the growth of breast cancer-derived lung metastases (Elia et al., 2019).

In addition, studies have found that increased metabolic reprogramming and glycolysis levels are associated with tumor progression (Li et al., 2022). Recent studies have shown that glycolysis/gluconeogenesis and carbon metabolism have been proven to be involved in the occurrence and development of lung cancer cells (He et al., 2022; Li et al., 2022). The tumor metabolic microenvironment plays an important role in tumor occurrence, development, invasion, and metastasis (García-Cañaveras et al., 2019; Dey et al., 2021). By analyzing these pathways, we found that these pathways are mostly related to the tumor metabolic microenvironment. Therefore, targeting tumor metabolism is of great significance for tumor immunity and tumor therapy.

In addition, we performed a prognostic analysis of the 10 CRGs and further constructed prognostic gene signature models based on these six prognostic CRGs (including GLS, CDKN2A, PDHA1, MTF1, LIPT1, and DLD) via LASSO Cox regression analysis. Previous studies have confirmed the prognostic value of ferroptosis-related genes (Gao et al., 2021), PANoptosis-related genes (Wang et al., 2022), and glycolytic-related genes (Zhang L et al., 2019) in LUAD. Surprisingly, the prognostic signature model of CRGs that we constructed demonstrated good potential in predicting the prognosis of patients with LUAD. This work is the first to evaluate the prognostic value of CRGs in LUAD, which provides more options for the prognostic analysis of LUAD. Environmental and metabolic pressure in the tumor microenvironment (TME) can play a key role in shaping tumor development by influencing matrix and immune cell composition, TME composition, and activation (Abou Khouzam et al., 2020). Therefore, we evaluated the relationship between these six CRGs’ prognostic label genes and immune infiltration, and we found that DLD was significantly correlated with most immune cell types, followed by PDHA1 and GLS. DLD, as a mitochondrial protein, plays an important role in energy metabolism in eukaryotes. It is involved in at least five multi-enzyme complexes and is a necessary component for the complex to complete the reaction. In addition, DLD, as a flavin protein oxidoreductase, accepts proton and electron-catalyzed disulfide bond formation with FAD as a co-group (Dai et al., 2019). Studies have confirmed that DLD is closely related to ferroptosis induced by cystine deprivation or import inhibition, and DLD inhibition can reduce lipid peroxidation and ferrous iron accumulation, thereby inhibiting ferroptosis suppression (Shin et al., 2020). Pyruvate dehydrogenase complex (PDC) plays a central role in carbohydrate metabolism, linking cytoplasmic glycolysis to the mitochondrial TCA cycle, and these regulatory serine residues in PDHA1 are structurally critical to enzyme activity (Echeverri Ruiz et al., 2021). In addition, PDHA1 is related to metabolic reprogramming in tumor diseases, such as esophageal cancer and gastric cancer (Liu et al., 2018; Liu L et al., 2019). Glutaminase (GLS) is a key enzyme involved in regulating glutamine metabolism and is reported to also play a crucial role in cancer development (Zhang J et al., 2019). These findings strongly suggest that CRGs may play an important role in LUAD. In particular, DLD is closely related not only to the prognosis of patients with LUAD but also to many immune cell types, which needs to be further verified by *in vitro* and *in vivo* experiments. We also constructed a DLD-related ceRNA regulatory network and identified the lncRNA UCA1/miR-1-3p/DLD axis. Previous studies have confirmed that lncRNA urothelial carcinoma-associated 1 (UCA1) is abnormally expressed in many cancers and has been confirmed as an oncogene (Huang et al., 2019; Yao et al., 2019; Yang et al., 2021). UCA1 promotes LUAD progression and cisplatin resistance, which may be a potential diagnostic marker and therapeutic target for patients with LUAD (Liu X et al., 2019; Fu et al., 2021). MiR-1-3p has been identified as a tumor suppressor in a variety of human cancers, including lung cancer (Jiao et al., 2018; Zhang H et al., 2019). Some studies have found that miR-1-3p expression in LUAD is decreased, whereas overexpressed miR-1-3p inhibits the proliferation, migration, and invasion of cancer cells (Miao et al., 2021). Excitedly, Differential expression of lncRNA UCA1, miR-1-3p and DLD in normal lung epithelial cells and LUAD cell lines was detected by RT-qPCR, and the results showed that lncRNA UCA1 and DLD were significantly overexpressed in LUAD cell lines, while miR-1-3p was on the contrary, and the above results further prove the accuracy of our bioinformatics results. In summary, these findings strongly suggested that the lncRNA UCA1/miR-1-3p/DLD axis may play an important role in the progression of LUAD.

Chinese herbal medicine is an important resource for discovering innovative medicines (Luo et al., 2019). The discovery of artemisinin further demonstrates the importance of TCM in innovative drug discovery (Ma et al., 2018). Therefore, we further screened the potential chemical components of TCM for the targeted regulation of DLD and performed molecular docking verification. As a result, we found nine potential TCM chemical components, namely, resveratrol, genistein, aristolochic acid I, cannabidiol, epigallocatechin gallate, fructose, phlorizin, quercetin, and triptonide. The molecular docking experiment results showed that all the nine potential TCM active ingredients had good binding activity to DLD, which further indicated that these ingredients may play an important role in the regulation of lncRNA UCA1/miR-1-3p/DLD axis. A large number of preclinical studies have shown that cannabidiol is an effective anticancer agent, whether used alone or in combination with other cannabinoids, chemotherapy, and radiation therapy (Seltzer et al., 2020). Resveratrol is a polyphenol compound originally isolated from the root of *Veratrum grandiflorum*. At present, a large number of studies have confirmed that resveratrol can inhibit the growth of LUAD cells, which can inhibit the expression of COX-2, arrest the cell cycle in the S phase, inhibit cell DNA synthesis, and inhibit the proliferation of A549 cells (Li et al., 2018; Li et al., 2019). At the same time, resveratrol can induce apoptosis and autophagy of lung adenocarcinoma cells by up-regulating the P53 level (Fan et al., 2020). When used in combination with gemcitabine (GEM), resveratrol has synergistic anticancer effects (Qin et al., 2020). Genistein exists in various plants for human and animal consumption, and it can inhibit the proliferation and induce apoptosis of A549 cells (Zhang et al., 2018). Moreover, genistein can downregulate lipid biosynthesis and inhibit the proliferation of human lung adenocarcinoma H460 cells (Hess and Igal, 2011). Cannabidiol can reduce Nrf-2 by targeting TRPV2 (transient receptor potential vanilloid-2), promote the production of reactive oxygen species (ROS), and inhibit the growth and metastasis of cisplatin-resistant NSCLC (Misri et al., 2022). Epigallocatechin gallate has been shown to induce the apoptosis of A549 cells by modulating ROS-mediated Nrf2/Keap1 signaling (Velavan et al., 2018). Quercetin can delay the development of LUAD and increase non-neoplastic weight gain in tumor oxidative stress mice (Albrecht et al., 2020). Other studies have found that quercetin nanoparticles can significantly reduce the viability of A549 cells, promote cell apoptosis, arrest the cell cycle in the G0/G1 phase, and reverse the drug resistance of A549 cells *in vitro* (Sun et al., 2020). In summary, these potential TCM chemicals may be an effective strategy for the treatment and improvement of the prognosis of LUAD. However, whether these potential active ingredients exert such effects by regulating DLD needs to be further verified by gene interference and other technologies.

In conclusion, we used a comprehensive bioinformatics strategy to elucidate the expression and prognosis of CRGs in LUAD. In addition, we constructed a cuproptosis-related prognostic gene signature model and found that DLD was most closely related to the prognosis and clinical and immune infiltration of LUAD. More importantly, we discovered a new potential ceRNA axis that regulates the LUAD process and identified the TCM active components that may regulate DLD. Interestingly, our experimental validation also preliminarily confirmed this bioinformatics screening result. This work provides a strong basis to interpret the prognostic value of CRGs and discover new therapeutic strategies. However, our study had some limitations. The validation of a prognostic signature related to CRGs should be further verified by more databases such as GEO, and future work should involve clinical samples, cell experiments, or animal experiments to verify our results.

## Data Availability

The original contributions presented in the study are included in the article/Supplementary Material, further inquiries can be directed to the corresponding authors.
