# Supplementary material for: Comprehensive bioinformatics analysis to identify a novel cuproptosis-related prognostic signature and its ceRNA regulatory axis and candidate traditional Chinese medicine active ingredients in lung adenocarcinoma
Source: Front Pharmacol. 2022 Aug 30;13:971867. doi: 10.3389/fphar.2022.971867 (PMC9468865; doi:10.3389/fphar.2022.971867)
Supplement: Supplementary file 1 [file DataSheet1.docx]

**Supplementary materials**

**For**

**Comprehensive bioinformatics analysis to identify a novel cuproptosis-related prognostic signature and its ceRNA regulatory axis and candidate traditional Chinese medicine active ingredients in lung adenocarcinoma**

*Shaohui Wang^1^, Nan Xing^2^, Xianli Meng^3^, Li Xiang^3*^*, *Yi Zhang^1*^*

^1^*State Key Laboratory of Southwestern Chinese Medicine Resources, School of Ethnic Medicine, Chengdu University of Traditional Chinese Medicine, Chengdu 611137, China*

*^2^State Key Laboratory of Southwestern Chinese Medicine Resources, School of Ethnic Medicine, Chengdu University of Traditional Chinese Medicine, Chengdu 611137, China*

*^3^State Key Laboratory of Southwestern Chinese Medicine Resources, Innovative Institute of Chinese Medicine and Pharmacy, Chengdu University of Traditional Chinese Medicine, Chengdu 611137, China*

**Corresponding authors:** Yi Zhang (zhangyi@cdutcm.edu.cn); Li Xiang (xianglydr@cdutcm.edu.cn)

**Figure Legend for Supplementary Data**

**Supplementary Figure 1.** A PPI interaction network of 10 CRGs was constructed by STRING database.

**Supplementary Figure 2.** Correlation between CTRP drug sensitivity and the six prognostic CRGs expression.

**Supplementary Figure 3.** The differential expression of the 27 lncRNAs in LUAD and normal lung tissues. ns, p≥0.05; *, p<0.05; **, p<0.01; ***, p<0.001.

**Supplementary Figure 4.** The OS curves of 8 lncRNAs in LUAD patients with low and high expression groups.

**FIGURE S1**


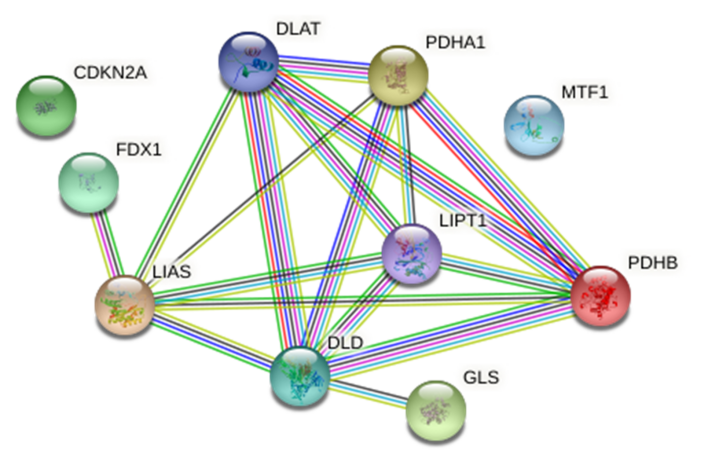


**FIGURE S2**

**
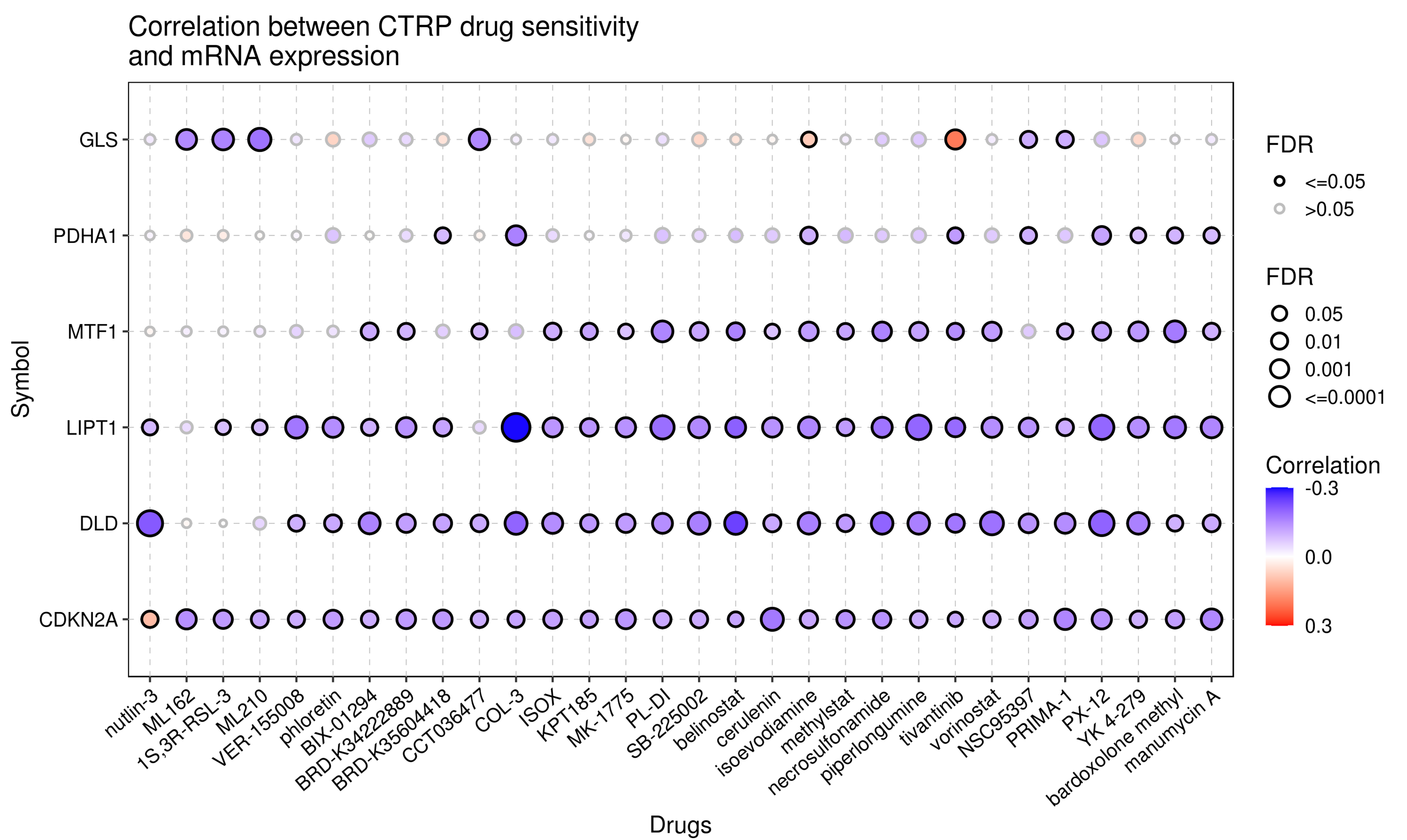
**

**FIGURE S3**

**
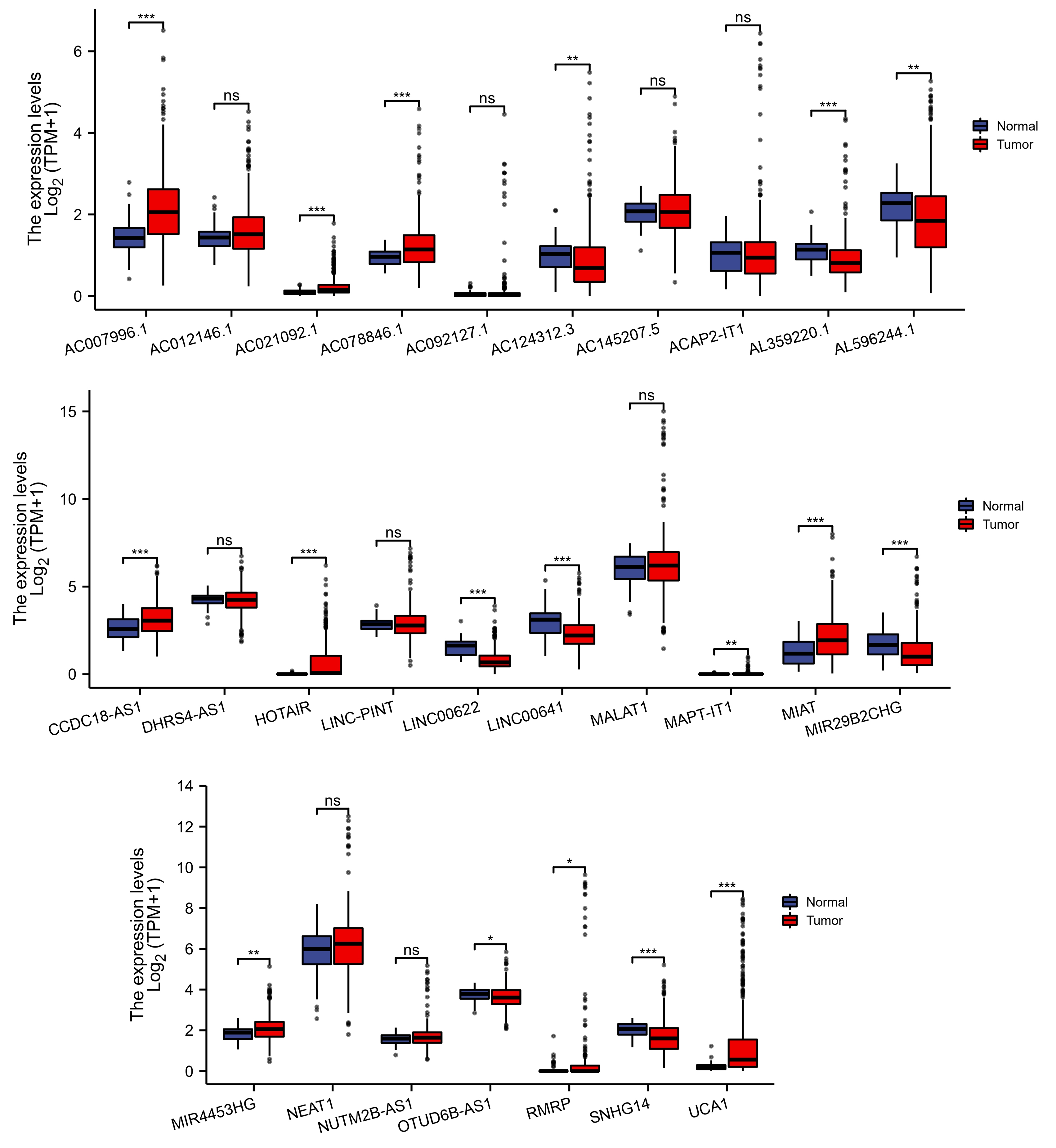
**

**FIGURE S4**


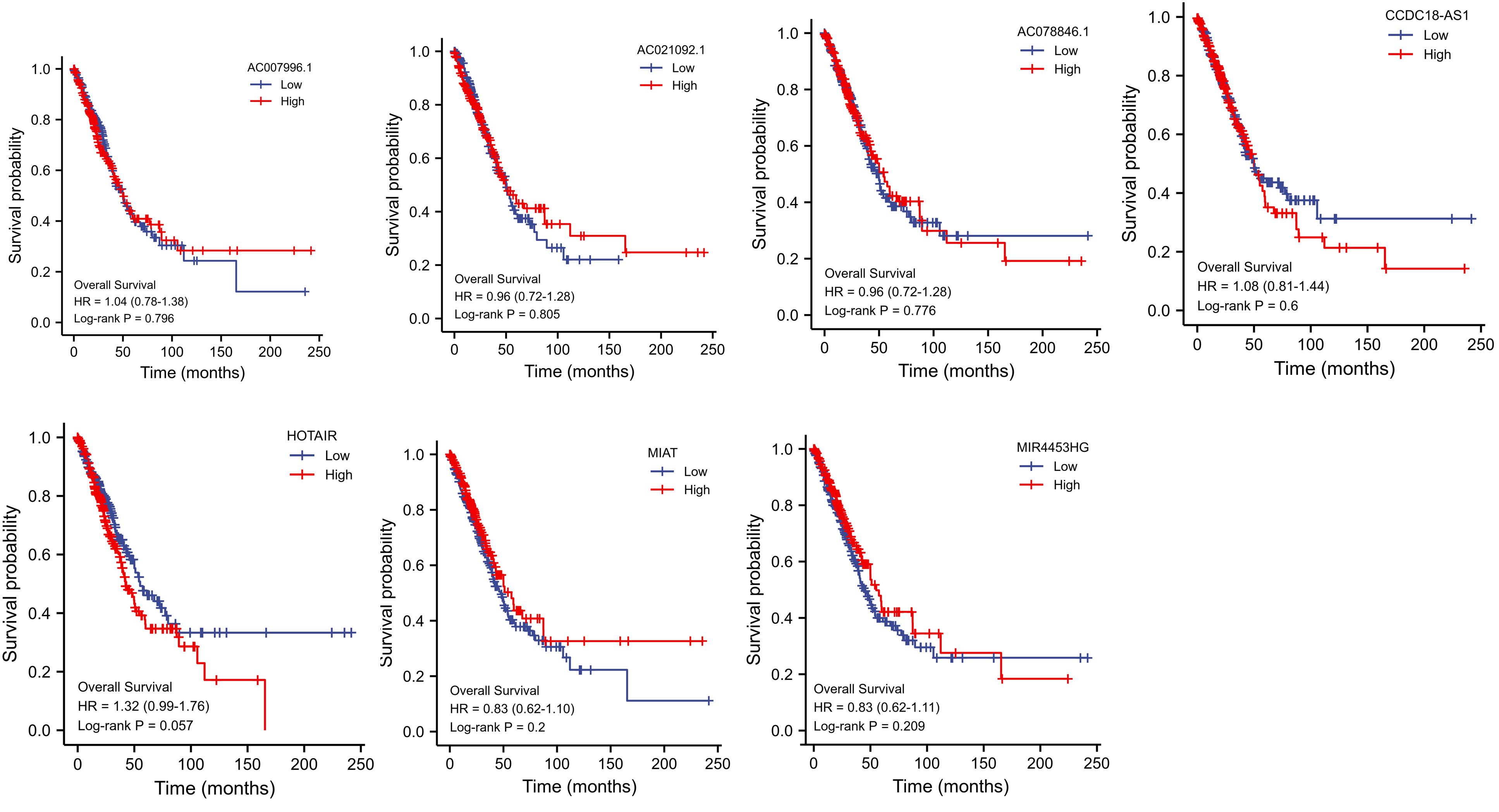


**Supplementary Table S1. The primer sequences.**

| **Primer** | **Sequence (5’-3’)** |
| --- | --- |
| miR-1-3p-F | GGCCTGGAATGTAAAGAAGT |
| miR-1-3p-R | CTCAACTGGTGTCGTGGAGTC |
| U6-F | CTCGCTTCGGCAGCACAT |
| U6-R | AACGCTTCACGAATTTGCGT |
| DLD-F | CCTTCTAAGGCTTTATTGAACAACT |
| DLD-R | GACTTGATTTTTGCCAGTTATCTTT |
| UCA1-F | GACAAACAACCTACAACCCTTAAGC |
| UCA1-R | TGTCCATTTCATGAGAGTAGGCTTG |
| GADPH-F | CATCATCCCTGCCTCTACTGG |
| GADPH-R | GTGGGTGTCGCTGTTGAAGTC |
